# The Wire Study—a protocol for a multi-stage feasibility study evaluating K-wire fixation of hand fractures in the UK

**DOI:** 10.1186/s40814-021-00858-4

**Published:** 2021-06-17

**Authors:** Justin Conrad Rosen Wormald, Mark Medhat Mikhail, Jeremy Neil Rodrigues, Sonya Gardiner, Theodore Pezas, Hawys Lloyd-Hughes, Fadi Issa, Abhilash Jain, Matthew David Gardiner

**Affiliations:** 1 Nuffield Department of Orthopaedics, Rheumatology and Musculoskeletal Sciences (NDORMS), Botnar Research Centre, University of Oxford, Oxford, OX3 7LD UK; 2 Department of Plastic, Reconstructive and Burns Surgery, Stoke Mandeville Hospital, Aylesbury, HP21 8AL UK; 3 Department of Plastic Surgery, Wexham Park Hospital, Frimley Health NHS Foundation Trust, Slough, SL2 4HL UK; 4 Warwick Clinical Trials Unit, University of Warwick, Coventry, CV4 7AL UK; 5 Department of Plastic, Reconstructive and Hand Surgery, Royal Free London NHS Foundation Trust, Pond St, Hampstead, London, NW3 2QG UK; 6 Department of Plastic, Reconstructive and Hand Surgery, St. George’s University Hospital NHS Trust, London, SW17 0QT UK; 7 Department of Plastic, Reconstructive and Hand Surgery, Guys and St. Thomas’ NHS Foundation Trust, London, SE1 7EH UK; 8 Nuffield Department of Surgical Sciences, University of Oxford, John Radcliffe Hospital, Oxford, OX3 9DU UK; 9 Department of Plastic, Reconstructive and Hand Surgery, Imperial College Healthcare Trust, London, W2 1NY UK

**Keywords:** Hand surgery, Trauma, Fracture

## Abstract

**Background:**

Hand fractures are common and sometimes require surgery to restore function. Placement of Kirschner wires (K-wires) is the most common form of surgical fixation. After placement, a key decision is whether to bury the end of a K-wire or leave it protruding from the skin (exposed). A recent systematic review found no evidence to support either approach and a national clinician and surgeon survey demonstrated further uncertainty. We aim to determine the design of a definitive randomised controlled trial assessing the cost and clinical effectiveness of buried versus exposed Kirschner wires for adults with metacarpal or phalangeal fractures.

**Methods:**

We will employ three methodologies: a national service evaluation of current clinical practice, patient and surgeon focus groups and a consensus meeting to finalise the protocol for a randomised controlled trial. For the service evaluation, all outcomes will be summarised using descriptive statistics overall and split by group (buried versus exposed K-wires). Information collected in the patient focus groups will be analysed thematically. The surgeon consensus meeting will address each part of the design in turn and through discussion agree a final protocol.

**Discussion:**

The study may be monitored, or audited in accordance with the current approved protocol, Good Clinical Practice (GCP), relevant regulations and standard operating procedures. The Chief Investigator will submit and, where necessary, obtain approval from the above parties for all substantial amendments to the original approved documents. A feasibility study report will be published by the Wire Study Steering committee. Additional members of the steering group and citable collaborators will be listed within the manuscript and their roles identified.

## Background

Hand injuries are common, with an estimated annual incidence of over 5 million injuries per year in the UK resulting in 250,000 operations, figures which continue to increase each year [1–5]. This is corroborated by Emergency Department statistics, which suggest that hand injuries account for over 20% of attendances [1, 2]. Furthermore, hand injuries are known to affect the younger, working population causing significant socioeconomic impact, as well as older patients where they disrupt daily activities of living and affect independence [6–8]. The optimal treatment of hand and wrist trauma was identified as one of the top ten priorities in the James Lind Alliance Priority Setting Partnership in September 2017 [9].

Fractures are one of the most common hand injures, accounting for approximately 19% of all fractures [2]. Most can be managed non-surgically, but a sizeable proportion undergo surgery. Closed reduction and percutaneous Kirschner wire (K-wire) fixation is the most common approach and is usually attempted within 2 weeks of injury under local, regional or general anaesthesia. Data from the British Society for Surgery of the Hand Open Fracture Audit indicates that around 70% of open hand fractures require K-wire fixation [10]. Following reduction of the fracture into an anatomical position, K-wires are passed across the fracture using a drill to hold the fragments in position. The surgeon then either shortens and bends the wire tip to leave it proud from the skin (exposed) or cuts them short and closes the skin over the wire (buried).

Whether K-wire ends should be buried or left exposed is an area of clinical uncertainty with substantial potential implications for the patient. A systematic review suggests a potentially higher rate of infection in exposed wires, but there is limited evidence to inform surgeon decision making [3]. We performed a national survey of clinicians and patients to further investigate this uncertainty. We found that those preferring to bury wires were motivated by reducing infection rates, whilst those preferring to leave them exposed cited ease of removal [4]. Surgeons also tailor their decision depending on patient characteristics. The clinical uncertainty is likely to have an impact on both patient and health economic outcomes, where the cost of removing a wire and the cost of infection must be weighed [5].

Before a randomised clinical trial can be conducted to answer this question, feasibility work is required. In particular, we need to know more about the variation in practice across the UK, so that we can ensure an RCT is feasible within the NHS. We also need to gather information about specific aspects of routine care, such as how and when patients are commonly followed-up, enabling us to minimise excess costs of a trial. Furthermore, information on the occurrence of infection will be useful in terms of sample size calculations for a future study. Once this information has been gathered, it can be used as a backdrop for feasibility discussions with both surgeons and patients to help refine the practical elements of a definitive RCT.

## Aims and objectives

### Aim

To determine the design of a definitive randomised controlled trial assessing the cost and clinical effectiveness of buried versus exposed K-wires for adults with metacarpal or phalangeal fractures in the hand.

### Objectives

- To conduct a prospective service evaluation to determine:
  - ○ The current clinical pathway of hand fracture patients undergoing K-wire fixation in the NHS
  - ○ The number of potentially eligible participants for a future RCT in K-wire fixation of hand fractures
  - ○ The number of K-wire fixation patients that attend routine follow-up
  - ○ The number of K-wire fixation patients that develop an infection to inform a sample size calculation for a definitive RCT
  - ○ The scope of the health economic evaluation needed in the trial to assess cost effectiveness
- To explore acceptability and feasibility of a future RCT in K-wire fixation of hand fractures by conducting patient and surgeon focus groups
- To develop the outline of a definitive RCT through a consensus workshop process with surgeons

## Methods

This is a mixed-methods feasibility study consisting of three stages: firstly, a national service evaluation of current clinical practice; secondly, patient and surgeon focus groups; and thirdly, we will bring these strands together in a consensus meeting to finalise the protocol for an RCT.

### Stage 1: National service evaluation

This will be a trainee-led prospective national multi-centre service evaluation coordinated by members of the WIRE ‘Steering Committee’. Trainees from across the UK and abroad will be invited to participate in the study through the Reconstructive Surgery Trials Network (RSTN). All centres that treat patients with hand trauma will be invited to participate, with the aim of maximising the scope of feasibility information and engaging with units for future participation in the definitive trial.

A local collaborator(s) will be identified at each institution (Trainee Lead). Collaborators will be responsible for identifying a supervising consultant surgeon (or equivalent) and obtaining local approvals at their unit. If no trainees are available within a unit, consultants, associate specialists, specialty doctors, hand therapists or research nurses will be encouraged to participate and enter data and will be eligible for the same acknowledgement. Support will also be sought from other professional associations, including the British Society for Surgery of the Hand (BSSH) and the British Association of Plastic, Reconstructive and Aesthetic Surgery (BAPRAS).

Instructions for local registration and data collection will be provided for collaborators. This will include a PowerPoint presentation and accompanying video which can be presented to the staff body in order to explain the study being carried out. Each team will enlist an appropriate number of collaborators given the number of expected patients to be identified during the study period. The name and contact details of the supervising consultant and trauma coordinator (if available) will also, with their permission, be documented to allow for continuity should contact be lost with the local Trainee Lead. The study will be piloted at the Oxford University Hospitals NHS Trust and Buckinghamshire Healthcare NHS Trust prior to roll-out to ensure ease, accuracy and confidentiality of data capture and inform recruitment of collaborators.

#### Study participants

Study participants for the service evaluation will be all patients deemed to require surgery, undergoing K-wire fixation of an acute fracture(s) of the hand; involving the metacarpals and/or phalanges who present to the participating unit. In patients with bilateral hand fractures or multiple fractures on one hand requiring K-Wire fixation; data will be entered for all fractures as per one case maintaining each patient as the unit of analysis.

##### Inclusion criteria

- Patients with primary K-wire fixation of open or closed fracture(s) of the metacarpals and/or phalanges
- Adults over 18 years old
- Male or female patients
- Primary surgery within or at 2 weeks post injury

##### Exclusion criteria

- Fixation strategies involving K-wires as part of an external fixator
- Nursing home residents, residential care residents or those serving custodial services
- Primary surgery more than 2 weeks post injury
- Concomitant wrist fracture or significant soft tissue injury

##### Data collection and entry

Data will be prospectively gathered and entered into the REDCap system. Login details will be supplied to collaborators once local permissions are confirmed in writing. Each patient will have a REDCap generated ID number assigned to them. This will be held locally alongside the patient hospital number. Only REDCap IDs will be held centrally. The full data dictionary is supplied in Appendix 1.

#### Clinical feasibility data

- Patient demographics
- Details of surgical procedures
- Details of antibiotic treatments
- Details of post-operative management and follow-up
- Details of K-wire removal timing and location of wire removal
- Use of patient reported outcome measures (PROMs)
- Details of discharge from hand surgery service
- Details of occurrence of post-operative infection
- Details of complications related to K-wire fixation
- Details of further surgery required

These data will be collected for when the K-wire is in situ, at the time of removal and up to 30 days.

##### Data validation and quality assurance

It is expected that participating hospitals will enter a complete data set for all patients identified as having metacarpal or phalangeal fractures managed with buried or exposed K-wires within the service evaluation period. Following data collection, approximately 5% of the centre’s data sets will be randomly selected. The consultant supervisor at each centre will be contacted and asked to independently validate a proportion of the data. If concordance is <80%, a further data sample will be randomly selected. If concordance remains <80%, the centre will be excluded from the analysis. If concordance is >95%, the consultant supervisor will be contacted to ensure the independence of the validation process and a further data sample will be randomly selected.

##### Data analysis plan

All data will be collated and managed centrally on a REDCap database. Data reports will be exported to an appropriate statistical software platform for analysis. We will perform descriptive analyses for all

### Stage 2: Patient and public involvement—focus groups

The patient focus groups aim to explore patient perspectives on the study in more detail. These will be semi-structured in nature. Likely topics to be discussed will include:

- Experience of K-wire fixation and postoperative rehabilitation
- Treatment expectations
- Consent process and recruitment to a trial
- Prioritising outcome measures
- Discussion of proposed trial design

The focus group will be facilitated by members of the study team with experience of running patient focus groups. They will not be part of the surgical community. Patients with experience of either buried or exposed K-wires will be invited to the focus groups. They will be recruited from regional hand surgery units and through advertising via the NIHR INVOLVE network. We will adopt maximum diversity sampling in selecting participants for the focus groups. The focus groups will have no more than eight participants in each session, and we aim to run three groups to achieve a thematic data saturation within funding and time constraints. The focus groups will not be recorded, but minutes will be recorded by a facilitator. The minutes will be circulated to the participants for verification. Patients will be reimbursed expenses in line with NIHR INVOLVE costings.

### Stage 3: Consensus meeting

Engaging the surgical community in agreeing the final details of the study protocol will be essential for a successful study. Surgical trials can be difficult to undertake, especially if surgeons remain uncertain about the objectives of the trial and treatment arms involved. The aim of the meeting is to achieve consensus within the surgical community regarding vital aspects of the trial design. We will invite the following stakeholders:

- Collaborators who have contributed to the service evaluation (stage 1)
- Patients and surgeons that take part in the focus groups (stage 2)
- Principal investigators of existing relevant research groups
- Members of key national societies:
  - ○ The British Society for Surgery of the Hand
  - ○ The British Association of Plastic, Reconstructive and Aesthetic Surgeons
  - ○ The British Orthopaedic Association
  - ○ The Orthopaedic Trauma Society

We will present outputs from the first two stages of this study and will discuss the following points, with an anonymous voting process to reach consensus:

- Types of injury to be included in the trial, e.g. open vs. closed
- Types of treatments to be included, e.g. single K-wire vs. external fixation frames
- Choice of primary outcome: infection, e.g. how it should be measured
- Other outcomes to be included, e.g. choice of PROM

The results of the consensus meeting will be collated and summarised by the steering group and published in an appropriate peer-reviewed journal.

### Study activities

#### Informed consent

No formal recording of consent is needed for this study. Stage 1 is a service evaluation collecting routine data and does not need patient consent. No changes in practice are occurring, no randomisation of treatment is occurring, and results will not be used to generalise to other patients. Patients and surgeons will imply consent by attending a focus group or consensus meeting.

#### Discontinuation or withdrawal from study

All participants have the right to withdraw from the study at any time. Those participants withdrawing will be offered two options: either to withdraw all of their data from the study (where it can be identified and removed) or to allow their data to be kept for inclusion in any analysis.

#### Definition of end of study

The end of the study will be when the consensus meeting has been completed, following completion of stages 1 and 2.

### Data management

#### Access to data

The WIRE Steering Committee and representatives from the sponsor or host institution will have direct access to the data.

#### Data recording and record keeping

Service evaluation data will be collected and managed using REDCap electronic data capture tools hosted at the Kennedy Institute of Rheumatology, University of Oxford. REDCap (Research Electronic Data Capture) is a secure, web-based application designed to support data capture for research studies, providing (1) an intuitive interface for validated data entry, (2) audit trails for tracking data manipulation and export procedures, (3) automated export procedures for seamless data downloads to common statistical packages and (4) procedures for importing data from external sources.

Data collection will remain in accordance with Caldicott II principles. Data for each patient will be pseudonymised using a unique alphanumeric study identification number. Local collaborators will be asked to keep an Excel spreadsheet linking hospital number to study number to prevent duplication of entries and to facilitate contemporaneous data entry at subsequent follow up appointments. This should be stored securely on the local hospital server according to local IT policies. No patient identifiable data will be transferred centrally. Minutes from the patient focus groups and clinician consensus meeting will be recorded by the trials team and kept on a secure University of Oxford network. Data from the WIRE Study will be kept for the duration of any definitive trial and archived according to the Sponsor’s requirement.

### Study management

Oversight of this study will be by the WIRE Steering Committee that will have wide representation from surgeons, trainees, the professional societies, patient representatives and those with experience of study management and statistics. This group is expected to meet twice a year but may also meet more frequently if necessary. Members of the WIRE Steering Committee will be assigned various tasks for day to day running of the study. It is expected that this will mainly work in a virtual/email forum facilitated through periodic group video conferencing to ensure writing and data analysis is adequately convened.

### Analysis

All analysis of pseudonymised data will occur centrally, with the support of statisticians and methodologists within the Surgical Interventional Trials Unit (SITU), University of Oxford.

#### Service evaluation

All outcomes will be summarised using descriptive statistics overall and split by group—buried versus exposed K-wires. We will examine histograms to determine whether it is parametric or non-parametric. Dichotomous, categorical and short ordinal outcomes will be summarised using counts and percentages. Continuous and long ordinal outcomes will be summarised by the mean with 95% confidence intervals (95%CIs), minimum and maximum (medians and IQRs will be reported for non-parametric data). A *p* value of 0.05 or less will be used to identify statistical significance for all analyses. Results for each participating hospital will be summarised and fed back to the local collaborators.

#### Patient focus group

Information collected in the patient focus groups will be analysed using Framework thematic analysis. New themes identified will be fed back into the subsequent focus groups so that they are incorporated into the proposed trial design.

#### Surgeon consensus meeting

A proposed trial design will be circulated in advance of the consensus meeting. It will be informed by all of the prior stages. The consensus meeting will address each part of the design in turn and through discussion agree a final protocol.

### Outcomes

Each feasibility outcome has been assigned green, amber and red criteria with respect to the feasibility of a definitive trial (Table 1) [11].

**Table 1:**
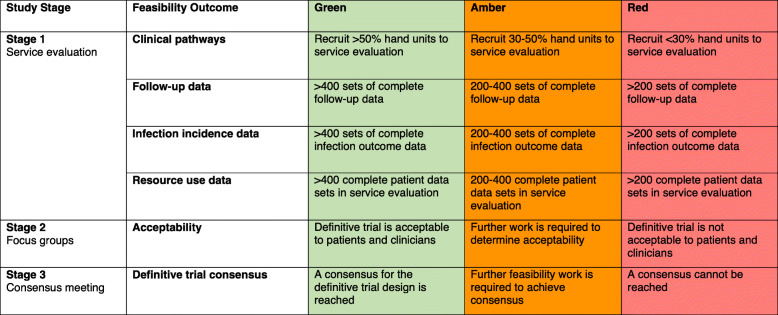
Progression criteria table

Description of the current clinical pathway of hand fracture patients undergoing K-wire fixation in the NHS. This will be successful if we are able to recruit over 50% of UK hand surgery units, allowing us to generate a representative picture of the majority of hand fracture clinical pathways. Engaging >50% of hand units will be a green indicator for a definitive trial. Collecting between 30 and 50% of units be an amber indicator and engaging <30% of units will be a red indicator. We will quantify the number of potentially eligible participants for a future RCT in K-wire fixation of hand fractures by assessing the throughput of patients through the above clinical pathways. We estimate that we will need c400 participants for a definitive trial. Therefore, to determine feasibility for follow-up rates, infections and resource use for a definitive trial we require complete data sets for at least 400 patients. This will be a green indicator for a definitive trial. Collecting between 200 and 400 complete data sets will be an amber indicator and attaining <200 will be a red indicator.

The patient and surgeon focus groups will determine acceptability of a future RCT in K-wire fixation of hand fractures. If the consensus is that the study is acceptable and feasible then this will be a green light indicator for a definitive trial. If further feasibility work is indicated, this will be an amber criterion. If the study is deemed neither feasible nor acceptable then this will be a red criterion. Lastly, the consensus meeting use outputs from stage 1 and 2 to determine whether a consensus can be reached for a definitive trial. If a consensus can be reached, then this will be a green criterion; if further work is needed, amber criterion; and if no consensus is possible, red criterion.

### Ethics

The study may be monitored, or audited in accordance with the current approved protocol, GCP, relevant regulations and standard operating procedures.

#### Declaration of Helsinki

The Chief Investigator will ensure that this study is conducted in accordance with the principles of the Declaration of Helsinki.

#### Guidelines for Good Clinical Practice (GCP)

The Chief Investigator will ensure that this study is conducted in accordance with relevant regulations and with GCP.

#### Approvals

The Chief Investigator will submit and, where necessary, obtain approval from the above parties for all substantial amendments to the original approved documents.

#### Participant confidentiality

Participants’ anonymity will be maintained. Clinician responses will be anonymous. Participants of the service evaluation will have identifiable details stored securely at the local site. These will be matched with a unique REDCap ID. The study will comply with the Data Protection Act and General Data Protection Regulation 2016/679.

#### Expenses and benefits

Patients attending the focus group will be offered high street vouchers for their time and reasonable travel expenses. Reasonable travel expenses for surgeons attending the workshop care will be reimbursed on production of receipts, or a mileage allowance provided as appropriate.

### Dissemination

All presentations and publications will be made on behalf of the WIRE Research Collaborative. The International Committee of Medical Journal Editors (ICMJE) criteria (www.icmje.org) for authorship are based on the following four criteria:

1. Substantial contribution to the conception or design of the work; or the acquisition, analysis or interpretation of the data for the work and
2. Drafting the work or revising it critically for important intellectual content and
3. Final approval of the version to be published and
4. Agreement to be accountable for all aspects of the work in ensuring that questions related to the accuracy or integrity of any part of the work are appropriately investigated and resolved.

The ICMJE states ‘when submitting a manuscript authored by a group, the corresponding author should specify the group name if one exists and clearly identify the group members who can take credit and responsibility for the work as authors. The by-line of the article identifies who is directly responsible for the manuscript and MEDLINE lists authors whichever names appear on the by-line. If the by-line includes a group name, MEDLINE will list the names of individual group members who are authors or who are collaborators, sometimes called non-author contributors, if there is a note associated with the by-line clearly stating that the individual names are elsewhere in the paper and whether those names are authors or collaborators.’ All citable collaborators will be asked to review the final manuscript before submission.

The key members of the steering group will be listed as authors in the by-line along with ‘on behalf of the WIRE Research Collaborative’. Additional members of the steering group and citable collaborators will be listed within the manuscript and their roles identified.

Local collaborators and hospitals will have ownership of their own data and will be able to present it locally if they wish. The final reports will be prepared in accordance with STROBE guidelines.

#### Criteria for citable collaborators status for WIRE SE

Recognition as a PubMed citable collaborator will occur when the following standards are met:

- Establish local registration and permissions
- Work as a team to ensure data accuracy
- Contribute ten data sets of consecutive patients during the study period
- Upload data sets that are at least 95% complete
- Answer any data queries promptly
- Consultant supervisors will be cited if data quality guaranteed and data validation on 5% of entries is completed on time

Citable collaborators will receive:

- A collaborator certificate outlining their contribution to the WIRE SE
- Collaborator acknowledgement on any presentations relating to the WIRE SE
- Their local data set for presentation
- PubMed citation on any subsequent publications relating to the WIRE SE. For example, see the WIRE Survey paper: https://www.ncbi.nlm.nih.gov/pmc/articles/PMC5977964/

Those not meeting the requirements of a PubMed-citable collaborator will be acknowledged contributors (see 11.2).

#### Acknowledged contributors for WIRE SE

Acknowledged contributors will include consultant surgeons who supported the project and other contributors who provided data but did not reach the threshold for citation. Acknowledged contributors will receive a certificate of participation for inclusion in their portfolios and acknowledgement in the manuscript.

## Data Availability

Not applicable.

## References

[1] Angermann P, Lohmann M (1993). Injuries to the hand and wrist. A study of 50,272 injuries. J Hand Surg Am.

[2] Clark DP, Scott RN, Anderson IW (1985). Hand problems in an accident and emergency department. J Hand Surg Am..

[3] Manley OWG, Wormald JCR, Furniss D (2019). The changing shape of hand trauma: an analysis of Hospital Episode Statistics in England. J Hand Surg Eur.

[4] O’Neill TW (2001). Incidence of distal forearm fracture in British men and women. Osteoporos Int..

[5] Warwick, D. Hand Surgery in the UK A resource for those involved in organising, delivering and developing services for patients with conditions of the hand and wrist. British Society for Surgery of the Hand (BSSH) - Report of a Working Party. 2017. Available at https://www.bssh.ac.uk/professionals/hand_surgery_in_the_uk.aspx. Accessed 1 Dec 2020.

[6] De Putter CE, Selles RW, Polinder S, Van Beeck EF (2012). Economic impact of hand and wrist injuries : J. Bone Jt Surg..

[7] Hunter JM, Cowen NJ (1970). Fifth metacarpal fractures in a compensation clinic population. A report on one hundred and thirty-three cases. J Bone Jt Surg.

[8] Trybus M, Lorkowski J, Brongel L, Hľadki W (2006). Causes and consequences of hand injuries. Am J Surg..

[9] James Lind Alliance Priorty Setting Partnership: Common conditions affecting the hand and wrist. Available at https://www.jla.nihr.ac.uk/priority-setting-partnerships/common-conditons-affecting-the-hand-and-wrist/. Accessed 10 Dec 2020

[10] Reconstructive Surgery Trials Network: BSSH Trauma Audits. Available at http://reconstructivesurgerytrials.net/clinical-trials/bsshaudit/. Accessed 15 Feb 2021

[11] Herbert E, Julious SA, Goodacre S (2019). Progression criteria in trials with an internal pilot: an audit of publicly funded randomised controlled trials. Trials..

